# CD47 Deficiency Ameliorates Ocular Autoimmune Inflammation

**DOI:** 10.3389/fimmu.2021.680568

**Published:** 2021-05-20

**Authors:** Yoko Okunuki, Steven J. Tabor, May Y. Lee, Kip M. Connor

**Affiliations:** Angiogenesis Laboratory, Department of Ophthalmology, Massachusetts Eye and Ear, Harvard Medical School, Boston, MA, United States

**Keywords:** uveitis, retina, autoimmune diseases, CD47 antigen, antigen-presenting cells

## Abstract

Autoimmune uveitis is a sight-threatening ocular inflammatory condition in which the retina and uveal tissues become a target of autoreactive immune cells. The CD47 is a ubiquitously expressed transmembrane protein which plays multiple roles in fundamental cellular functions including phagocytosis, proliferation, and adhesion. Signal regulatory protein alpha (SIRPα), one of the CD47 ligands, is predominantly expressed in myeloid lineage cells such as dendritic cells (DCs) or macrophages, and CD47-SIRPα signaling pathway is implicated in the development of autoimmune diseases. Our current study demonstrates how CD47 depletion is effective in the prevention of experimental autoimmune uveitis (EAU), an animal model of human autoimmune uveitis, in animals deficient of CD47 (*CD47^-/-^*). Systemic suppression of SIRPα^+^ DCs in animals deficient in CD47 resulted in the inability of autoreactive CD4^+^ T cells to develop, which is crucial to induction of EAU. Of interest, retinal microglia, the resident immune cell of the retina, express SIRPα, however these cells were not operative in EAU suppression in response to CD47 depletion. These results identify CD47 as a significant regulator in the development of SIRPα^+^ DCs that is vital to disease induction in EAU.

## Introduction

Autoimmune uveitis, which occurs in a variety of diseases, including Bechet’s disease, sarcoidosis, and Vogt–Koyanagi–Harada disease, among many others, is a sight-threatening ocular inflammatory disease (1–4). Although autoimmune uveitis covers a range of different clinical entities, autoimmunity against the retina and the uveal tissues is thought to be fundamental to its pathogenesis (5).

Experimental autoimmune uveitis (EAU) is an animal model of human autoimmune uveitis, and is widely used to delineate the pathophysiological processes of ocular autoimmunity and develop new approaches to treat patients (6). EAU is induced by immunization against retinal antigens such as interphotoreceptor retinoid-binding protein (IRBP), which is a major component of photoreceptor outer segments. IRBP immunization thus targets the immune response to photoreceptors (7, 8).

Uveitogenic antigen-specific CD4^+^ T-cells are crucial effector cells that drive inflammation and tissue damage after being activated by dendritic cells (DCs) (9). Systemic immunization with IRBP and additional adjuvants leads to the antigen uptake by systemic DCs which in response activate antigen specific CD4^+^ T cells by presenting the antigen *via* MHC class II (MHC-II) in the peripheral lymphoid tissues.

The activated antigen specific CD4^+^ T cells proliferate and invade the retina following the breakdown of blood retinal barrier. Our group have shown that microglia facilitate autoreactive CD4^+^ T cell extravasation into the retina concurrent with other activated immune cell populations such as antigen presenting cells (APCs) (10). These immune cells are re-activated by cross-reactive retinal autoantigens and subsequently substantial inflammation ensues (11). There are multiple receptors, chemokines, and cytokines suggested to be involved in the development of autoimmune uveitis. Recently CD47-signal regulatory protein alpha (SIRPα) interactions have been implicated in induction of T cell-mediated inflammation (12, 13), and thus we sought to assess the role of this signaling axis in a pre-clinical model of uveitis that is largely driven by T-cell activation.

CD47, also known as integrin-associated protein (IAP), is an immunoglobulin-like protein ubiquitously expressed on the surface of virtually all cell types including erythrocytes and immune cells (14, 15). CD47 exerts pleiotropic functions by interacting with its ligands including SIRPα, thrombospondin-1 (TSP-1), and various integrins (16). Unlike the ubiquitous expression of CD47, SIRPα is expressed on limited number of cell populations including myeloid cells (monocytes, macrophages, DCs, and granulocytes) and neurons (17). Engagement of CD47 with SIRPα^+^ phagocytes, such as DCs and macrophages, provides a down regulatory signal that inhibits host cell phagocytosis by SIRPα^+^ phagocytes (18). Therefore, CD47 functions as a marker of self and is called the “don’t-eat-me” signal (19). This regulatory mechanism of phagocytosis initiated by CD47-SIRPα^+^ interaction is well studied in red blood cell elimination by splenic macrophages (18) and in cancer immunity (19).

However, evidence from recent studies suggests that CD47-SIRPα interactions are key for efficient induction of T cell–mediated inflammation (20–22). One mechanism by which this is thought to occur is mediated through the interaction between SIRPα on DCs and CD47 on T cells to promote antigen presentation and T cell proliferation (23). The CD47-SIRPα interaction appears to activate and direct the migration of SIRPα^+^ cells including neutrophils, melanoma cells and monocytes (24–26). Interestingly, it has been reported that SIRPα^+^ monocytes interact with CD47 on brain endothelial cells to facilitate their transmigration across the blood brain barrier (26). Similarly, in an *in vivo* study of inflammatory bowel disease, it was demonstrated that interaction between SIRPα^+^ DCs and CD47^+^ T cells is required for DCs to exert their migration and antigen presentation abilities (12). Therefore, it is likely that CD47-SIRPα interaction contributes to antigen presentation and T cell activation in the induction of a T cell mediated inflammatory diseases. Thus, this CD47-SIRPα interaction between DCs and CD4^+^ T cells has the potential to be an important mechanism in the pathogenesis of autoimmune diseases. Here we demonstrate that systemic expression of CD47 is essential in induction of autoimmune uveitis through promotion of development of SIRPα^+^ DCs which has a crucial role in the priming of autoreactive CD4^+^ T cells.

## Materials and Methods

### Animals and Reagents

Female animals were used for all of the experiments otherwise mentioned. All animal experiments followed the guidelines of the ARVO Statement for the Use of Animals in Ophthalmic and Vision Research and were approved by the Animal Care Committee of the Massachusetts Eye and Ear Infirmary. C57BL/6J mice (stock no. 00664) were purchased from Jackson Laboratories (Bar Harbor, ME). Standard laboratory chow was fed. Mice were allowed free access to food and water in a climate-controlled room with a 12-hour light/12-hour dark cycle. All mice used for experiments were 7-10 weeks old. For anesthesia, intraperitoneal injection of 250 mg/kg of 2,2,2-tribromoethanol (Sigma-Aldrich Corp., St. Louis, MO) was used for survival procedures, and 400 mg/kg was used for non-survival procedures. High performance liquid chromatography-purified human interphotoreceptor retinoid binding protein peptide 1**-**20 (IRBP-p) was purchased from Biomatik (Wilmington, DE). Complete Freund**’**s Adjuvant and *Mycobacterium tuberculosis* H37Ra were purchased from Difco (Detroit, MI).

### Induction of EAU

For active induction of EAU, 200 µg of IRBP-p 1-20 was emulsified in Complete Freund**’**s Adjuvant (1:1 w/v) containing additional 5 mg/ml *M. tuberculosis* H37Ra. On day 0, 200 µl of the emulsion was injected subcutaneously in the neck (100 µl), one footpad (50 µl) and the contralateral inguinal region (50 µl). Concurrent with immunization, 1 µg of purified Bordetella pertussis toxin (Sigma**-**Aldrich) was injected intraperitoneally.

Adoptive transfer EAU was induced as previously described (10). Briefly, donor mice were immunized as described above, and SPs and draining lymph nodes (LNs) were collected at 14 days after immunization. Lymphocytes from spleens (SPs) and draining LNs were cultured in the presence of 10 µg/ml IRBP-p and 10 ng/ml IL-23 (R&D systems, Minneapolis, MN) for 72 hours in RPMI 1640 supplemented with 10% FBS, 10 mM HEPES, 1 x nonessential amino acid, and 1 mM sodium pyruvate (ThermoFisher, Waltham, MA), and 50 µM 2-mercaptoethanol, 100 U/ml penicillin and 100 µg/ml streptomycin (Sigma-Aldrich). IL-23 was added in the culture in order to induce effector T cells efficiently (27). The non-adherent cells in suspension were transferred to new dishes on day one and two of culture. After 3 days, activated lymphocytes were purified by gradient centrifugation on Histopaque 1083 (Sigma-Aldrich) and counted. The cells were injected intraperitoneally in 0.2 ml of PBS into recipient mice (4 × 10^7^ cells/mouse).

### Assessment of EAU

Fundus images were observed using a Micron IV retinal imaging microscope (Phoenix, Pleasanton, CA) and the clinical score of active inflammation was graded in a blinded manner on a scale between 0-4 in half-point increments as described previously (28) with brief modification. Briefly, trace chorioretinal lesions and minimal vasculitis were score as 0.5. Mild vasculitis with small focal chorioretinal lesions (≤5) were scored as 1. Severe vasculitis with multiple chorioretinal lesions (>5) were scored as 2. Pattern of linear chorioretinal lesion, subretinal neovascularization and hemorrhages were scored as 3. Inflammation with large retinal detachment and severe hemorrhages were score as 4. For histological assessment, enucleated eyes were fixed in a buffer of 70% methanol and 30% acetic acid. The fixed tissues were embedded in paraffin and processed. Sections of 5 µm were cut and stained with hematoxylin and eosin (H&E). The severity of EAU in each eye was scored on a scale between 0-4 in half-point increments in a blinded manner as described previously (29). Briefly, focal non-granulomatous, monocytic infiltration in the choroid, ciliary body and retina were scored as 0.5. Retinal perivascular infiltration and monocytic infiltration in the vitreous were scored as 1. Granuloma formation in the uvea and retina, the presence of occluded retinal vasculitis, along with photoreceptor folds, serous retinal detachment and loss of photoreceptor were scored as 2. In addition, the formation of granulomas at the level of retinal pigment epithelium and the development of subretinal neovascularization were scored as 3 and 4 according to the number and the size of the lesions (30). The average of scores from both eyes was determined as the score of the animal.

### Delayed Hypersensitivity Measurement

Antigen-specific delayed hypersensitivity was measured as previously described (31). On day 19 after immunization, mice were injected intradermally with 10 µg of IRBP-p suspended in 10 µl PBS into the pinna of one ear. Ear swelling was measured after 48 hours using a micrometer (Mitutoyo, Tokyo, Japan). Delayed hypersensitivity was measured as the difference in ear thickness before and after challenge. Results were expressed as: specific ear swelling = (48-hour measurement – 0-hour measurement) for test ear – (48-hour measurement – 0-hour measurement) for control ear.

### Flow Cytometric Analysis of Lymph Nodes and Spleens

Cervical, axillary, and inguinal LNs and SPs were harvested from naive mice and EAU mice. Single cell suspensions were blocked with anti-mouse CD16/32 mAb (ThermoFisher). Dead cells were stained with LIVE/DEAD™ fixable dead cell stain kit (blue or violet) (ThermoFisher) or Zombie Fixable Viability Kit (UV or NIR) (Biolegend). The following anti-mouse antibodies were used for staining: CD4 (clone: GK1.5), CD8 (53-6.7), CD19 (6D5), CD11c (N418), CD11b (M1/70), CD45 (30-F11), CD3 (17A2), CD44 (IM7), CD62L (MEL-14), CD25 (PC61.5), CD80 (16-10A1), CD86 (GL-1), MHC-II (M5/114.15.2), NK1.1 (PK136), and Ly6G (1A8) were purchase from BioLegend (San Diego, CA). CD11c-BB515 (N418) and SIRPα (P84) was purchased from BD Bioscience. Flow cytometric data were acquired on a CytoFlex S (Beckman Coulter, Brea, CA). Single color staining and fluorescence minus one (FMO) controls were used for accurate compensation and gating. Acquired data was analyzed using FlowJo 10.5.3 (Ashland, OR).

### Flow Cytometric Analysis of Retinal Microglia

For flowcytometric analysis of retinal microglia, mice were first perfused with PBS, and then the eyes were enucleated and the retinas were collected. Both retinas of each animal were combined and incubated for 45 min at 37°C in HBSS containing 10% FBS, 10 mM HEPES, 0.7 mg/ml calcium chloride (Sigma-Aldrich), 1 mg/ml collagenase D, and 0.1 mg/ml DNase I (Roche). After the incubation, retinas were homogenized by gentle trituration by pipetting and transferred to a FACS tube by passing through a 40 µm cell strainer. The samples were centrifuged at 350 rcf for 5 min, re-suspended in FACS buffer, and further centrifuged at 50×g for 1 min to precipitate debris (32). Supernatant containing cells was collected and used for subsequent staining after washing with FACS buffer a couple of times. The cell suspensions were blocked with anti-mouse CD16/32 mAb. Dead cells were stained with DAPI and excluded from the analysis. Microglia were identified as a CD11b^+^CD45^low^ population and SIRPα positive cells were evaluated.

### Lymphocyte Proliferation Assay

Cells were isolated from draining LNs and SPs, and suspended at 2 × 10^5^ per 200 µL medium in 96-well flat-bottom plates. The cells were cultured in triplicate in the presence of various concentration of IRBP-p or medium alone. The last four hours of 72 hours culture, 100 µl of supernatant in the culture medium was removed and 10 µl of Cell Counting Kit-8 (Sigma-Aldrich) was added each well and the cells were incubated for 4 hours. At the end of culture, the viable cell numbers in each well were measured as the absorbance (450 nm) of reduced WST-8 (33).

For mixed lymphocyte proliferation assay of CD11c^+^ DC priming of the CD4^+^ T cell, first CD4^+^ T cells were isolated from LNs by MACS system (Miltenyi Biotec, Bergisch Gladbach, Germany) using CD4^+^ T cell isolation kit. CD11c^+^ DCs were isolated from SPs using CD11c MicroBeads UltraPure. Isolated CD4^+^ T cells were added to a solution containing CellTrace™ Far Red (CTFR)(ThermoFisher) in PBS at 37°C for 20 min following the manufacture instruction (34). Aliquots of CTFR-labeled CD4^+^ T cells (2× 10^5^) in U-bottom 96-well plates were co-cultured with isolated CD11c^+^ DC (1× 10^5^) in 200 µl/well in the presence of 10 µg/ml IRBP-p for 3 days. The frequency and number of proliferated CD4^+^ T cell in each sample was determined by diluted CTFR gated on live CD3^+^CD4^+^ cells.

### Immunohistochemistry of Whole-Mount Retinas

Anesthetized mice were perfused with 20 mL of 1X PBS and eyes were enucleated. Whole eyes were fixed in 4% paraformaldehyde in 2X PBS for 45 minutes and then transferred to 2X PBS on ice for 15 minutes. Following the removal of the lens, retinas were isolated and prepared with four radical incisions. Retinas were subsequently transferred to ice cold methanol and stored for further use at -80°C. For immunohistochemistry, retinas were washed in 1X PBS three times and blocked overnight in blocking buffer (5% goat serum, 0.03% triton-X100, 0.2% BSA in 1X PBS). Retinas were briefly washed in 1X PBS and then incubated with primary antibodies: rabbit anti-P2ry12 (1:500; a gift from H. Weiner, Brigham and Women’s Hospital) and rat anti-SIRPα (1:500 Clone: P84 from Biolegend) in blocking buffer overnight at 4°C. Retinas were then washed four times in 1X PBS. Retinas were incubated with secondary antibodies goat anti-rabbit 594 (1:500), and goat anti-rat 647 (1:500) from ThermoFisher in blocking buffer overnight at 4°C. Whole-mounts were then washed four times in 1X PBS and mounted using anti-fade medium (Permaflour, Thermo Fisher Scientific, Waltham, MA). Images were taken on a Lecia Confocal Sp8.

### Quantification and Statistical Analysis

Data are presented as mean ± standard error of the mean (SEM). Differences between two groups were analyzed using an unpaired Student’s *t*-test or Mann-Whitney test. Multiple-group comparison was performed by one-way ANOVA followed by Tukey’s multiple comparison test. All statistical analyses were performed using graphing software (Prism 6, GraphPad Software, Inc., La Jolla, CA). Significance levels are marked *P<0.05; **P<0.01; ***P<0.001; ****P<0.0001 in figures.

## Results

### Loss of CD47 Suppresses Autoimmune Uveitis

**Figure 1:**
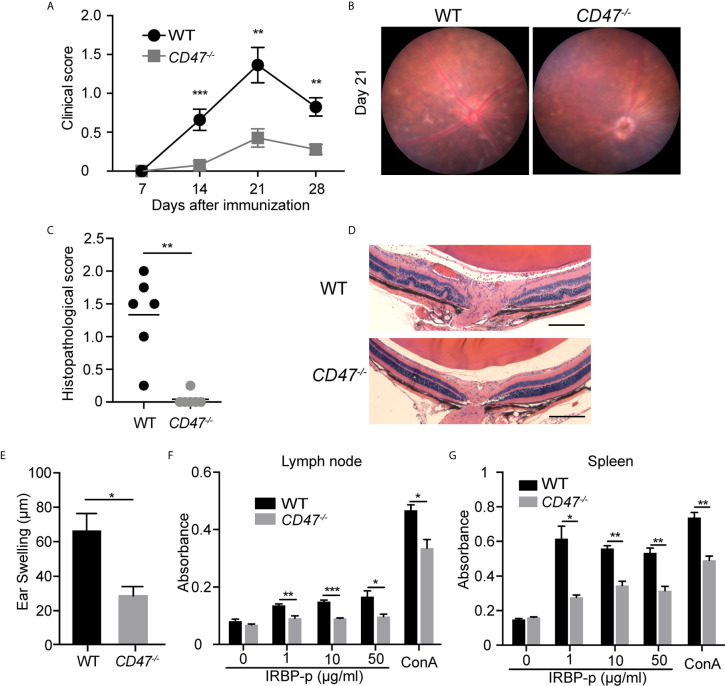
CD47 deficient mice are protected from autoimmune uveitis. Female WT and *CD47^-/-^* mice were immunized with IRBP-p and the animals were evaluated for **(A–D)** severity of retinal inflammation and **(E–G)** systemic immune responses against the IRBP-p. **(A)** Time course of EAU clinical scores were evaluated by fundus observation (n =10-11 mice per group). **(B)** Representative retinal fundus images on day 21. **(C)** Histopathological EAU score was evaluated on day 21 (n = 6 mice per group). **(D)** Representative histopathological (H&E staining) images (Scale bars: 200 μm). **(A, C)** Data were analyzed by Mann–Whitney *U* test. **(E)** Delayed hypersensitivity, as determined by ear swelling, was evaluated on day 21 (n=10-11). **(F, G)** Cell proliferation was evaluated by using the cells isolated from lymph nodes and spleens on day 21. The cells were cultured in triplicate for 3 days in the presence of IRBP-p (10 μg/ml), Con A (1 μg/ml), or medium only (n = 5 mice per group). **(E–G)** Data were analyzed by Student’s *t* test. Data are expressed as mean ± SEM. *P < 0.05; **P < 0.01; ***P < 0.001.

To evaluate the overall contribution of CD47 in development of autoimmune uveitis, we assessed disease severity in mice with a systemic deficiency of CD47. CD47 knockout (*CD47^-/-^*) mice were utilized and EAU was induced by immunizing the animals with IRBP-peptide 1–20 (IRBP-p) in both knockout animals and littermate wild type (WT) controls. Ocular inflammation associated with EAU was observed from day 7 to day 28 after immunization. Clinical assessment of these animals by fundoscopic examination found that EAU was significantly suppressed in female *CD47^-/-^* mice throughout the course of disease, whereas the control animals developed significant retinal inflammation (**Figures 1A, B** **)**. Similarly, *CD47^-/-^* male mice were assessed and found to exhibit an equivalent degree of EAU suppression as their female counterparts (**Supplemental Figure S1**). Female animals had been conventionally used in studies of EAU (7, 30, 35–40), however there did not seem to be an obvious difference in susceptibility to EAU between males and females (29, 41). Since male *CD47^-/-^* mice exhibited the same degree of disease inhibition as females, female mice were utilized for the remainder of the study. Histological evaluation of EAU disease severity at the peak of inflammation in this model, day 21, further confirmed that CD47 deficiency significantly suppressed infiltrating inflammatory cells and retinal damage (**Figures 1C, D** **)**. Although autoimmune uveitis is a condition observed locally in the eye, systemic immune activation against the retinal auto-antigen leads to ocular inflammation. Therefore, we next evaluated the systemic immune response against the immunized peptide by (i) IRBP-specific delayed hyper-sensitivity *in vivo*, and (ii) IRBP-specific and nonspecific (Concanavalin A-induced) lymphocyte proliferation *in vitro*. We found that the systemic IRBP-specific immune response was suppressed in *CD47^-/-^* mice both *in vivo* and *in vitro*. The induction of delayed hypersensitivity in the ear against IRBP-p was significantly reduced in *CD47^-/-^* mice (**Figure 1E**) compared to control animals. Moreover, IRBP-specific and non-specific *in vitro* lymphocyte proliferation using lymph node (LN) cells and spleen (SP) cells, isolated from mice with EAU at 14 days (**Supplemental Figure S2**) and 21 days (**Figures 1F, G** **)** post immunization was significantly reduced in *CD47^-/-^* mice. These results indicate that the systemic immune response against the immunized peptide was suppressed in *CD47^-/-^* mice and that the suppressive effects of EAU observed in *CD47^-/-^* mice involve the suppression of systemic immune cell activation in response to IRBP-p autoantigen, not locally in the retina.

### Suppression of EAU in CD47 Deficient Mice Was Not Regulated Within the Retinal Microenvironment

**Figure 2:**
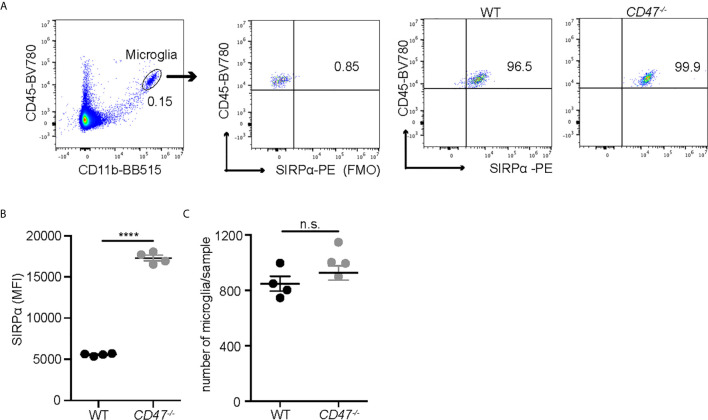
SIRPα expression on retinal microglia is upregulated in naïve *CD47^-/-^* mice. Retinal microglia are isolated from naïve WT and *CD47^-/-^* mice. Both retinas from one mouse were combined and processed as one sample. Retinal single cell suspensions were gated on live cells (DAPI^-^). SIRPα expression on microglia (CD11b^+^CD45^low^) was examined by flow cytometry. **(A)** The gating strategy of microglia and SIRPα, and representative sample from each group. **(B)** MFI of SIRPα on microglia. **(C)** The number of microglia per sample (contains two retinas). Data were analyzed by Student’s *t* test and expressed as mean ± SEM. ****P<0.0001. n.s., not significant. n=4 mice per group.

EAU was suppressed in animals that lack systemic expression of CD47 and our data assessing the delayed hypersensitivity response and immune cell proliferation against the antigen strongly implicated the suppression of systemic immunity as the mechanism of EAU inhibition. However, CD47 as well as its ligands, especially TSP-1, are expressed within the retina (42). In addition, we identified that microglia, the resident immune cells in the retina, express SIRPα under normal conditions (**Supplemental Figure S3**) and that microglial expression of SIRPα in *CD47^-/-^* mice was higher than in WT mice, although total microglial numbers in the retina was not largely affected by the loss of *CD47* (**Figure 2**). It has been demonstrated that microglia are actively involved in the development of EAU especially in the early stage of inflammation (10). Therefore, it was possible that locally expressed CD47 and its ligands play a direct role in the disease course of EAU. To examine this possibility, we utilized the adoptive transfer model of EAU.

**Figure 3:**
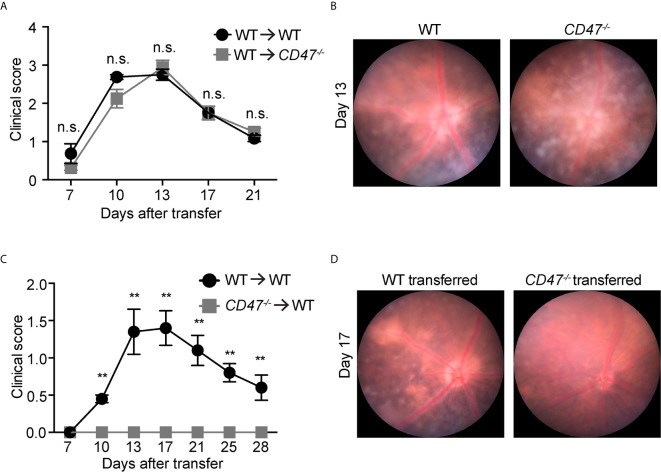
CD47 deficiency in systemic immune cells but not locally in the eye is protective for autoimmune uveitis. Time course of clinical score and representative fundus images using adoptive transfer EAU model. **(A, B)** WT donor mice were immunized and the primed cells were transferred to naive WT or *CD47^-/-^* recipient mice. **(A)** Time course of EAU clinical scores were evaluated by fundus observation (n =4 mice per group) **(B)** Representative retinal fundus images on day 13. **(C, D)** WT and *CD47^-/-^* donor mice were immunized and the primed cells were transferred to naive WT recipient mice. **(C)** Time course of EAU clinical scores were evaluated by fundus observation (n=5-6 mice per group) **(D)** Representative retinal fundus images on day 17. Data were analyzed by Mann**–**Whitney *U* test and expressed as mean ± SEM. **P<0.01; n.s., not significant.

In this model, IRBP-reactive immune cells were transferred from donor immunized mice into naive recipient mice, subsequently donor cells induced EAU in recipient animals without additional stimulation. Here, primed cells from IRBP-immunized WT donor mice were adoptively transferred into naïve WT or naïve *CD47^-/-^* recipient mice. This approach excluded any potential effects of systemic immune suppression in *CD47^-/-^* recipient mice on the systemic cell priming stage and will allow us to elucidate the involvement of the retinal specific immune response in recipient mice. We found that significant inflammation in both recipient groups (naïve WT and naïve *CD47^-/-^*) with a comparable severity of disease induction (**Figures 3A, B** **)**, indicating that expression of CD47 on primed circulating immune cells significantly affects the disease course of EAU. Strengthening this finding was the converse experiment, when primed cells from *CD47^-/-^* mice were transferred to naïve WT mice, the recipient animals did not develop EAU (**Figures 3C, D** **)**. In this approach, the systemic immune response of donor mice (WT or *CD47^-/-^* mice) defined the severity of EAU in the recipient mice. Therefore, these results further indicate that systemic immune suppression is the fundamental mechanism of decreased EAU severity in *CD47^-/-^* mice.

### Loss of CD47 Decreases Systemic CD11b^+^ Populations

**Figure 4:**
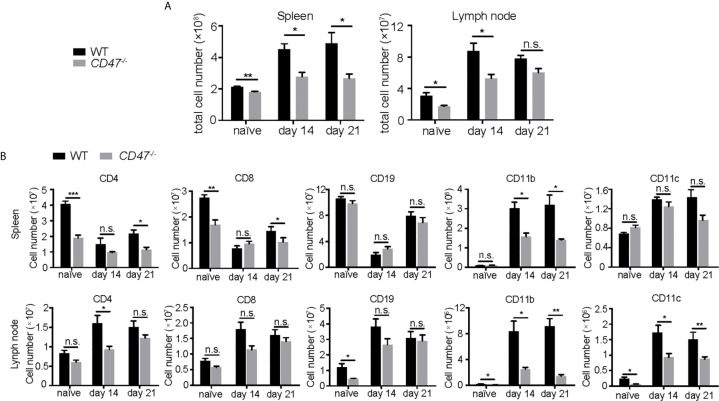
The number of T cells, macrophages, and DCs are decreased in CD47 deficient mice with autoimmune uveitis. Spleen and lymph node cells from WT and *CD47^-/-^* mice were collected before (naive), and 14 days and 21 days post immunization. **(A)** Total cell number in spleens and lymph nodes. **(B)** CD4^+^, CD8^+^, CD19^+^, CD11b^+^, CD11c^+^ cells in spleens and lymph nodes were detected by flowcytometry and cell number of each population was calculated. The cells were gated on live CD45^+^CD3^+^ cells to detect CD4^+^ T cells and CD8^+^ T cells. CD45^+^CD3^-^ population was further gated on CD19, CD11b, and CD11c. Data were analyzed by Student’s *t* test and expressed as mean ± SEM. *P<0.05; **P<0.01; ***P<0.001. n.s., not significant. n=4 mice per group.

To begin to identify the key mechanism by which EAU is suppressed from the loss of CD47, we examined how specific immune cell populations in peripheral lymphoid organs were affected due to the loss of CD47. We first examined major immune cell populations known to be involved in the disease pathogenesis of EAU (CD4^+^ T cells, CD8^+^ T cells, CD19^+^ B cells, CD11b^+^ macrophages, CD11c^+^ DCs) in SPs and LNs by flow cytometry, before (naïve) and after EAU induction (days 14 and 21). *CD47^-/-^* mice had fewer cell numbers in SPs and LNs than WT mice before and after EAU (**Figure 4A**). Further analysis of each specific population revealed that CD4^+^ and CD8^+^ T cells were mildly decreased in *CD47^-/-^* mice, whereas the number of CD19^+^ B cells remained unchanged. On the contrary, CD11b^+^ cells in both SPs and LNs and CD11c^+^ cells in LNs were significantly decreased during EAU in *CD47^-/-^* mice (**Figure 4B**, **Supplemental Figure S4**). This indicated that macrophages (CD11b^+^) and DCs (CD11c^+^) are directly regulated by CD47 during EAU and that T cells are regulated to a lesser extent.

**Figure 5:**
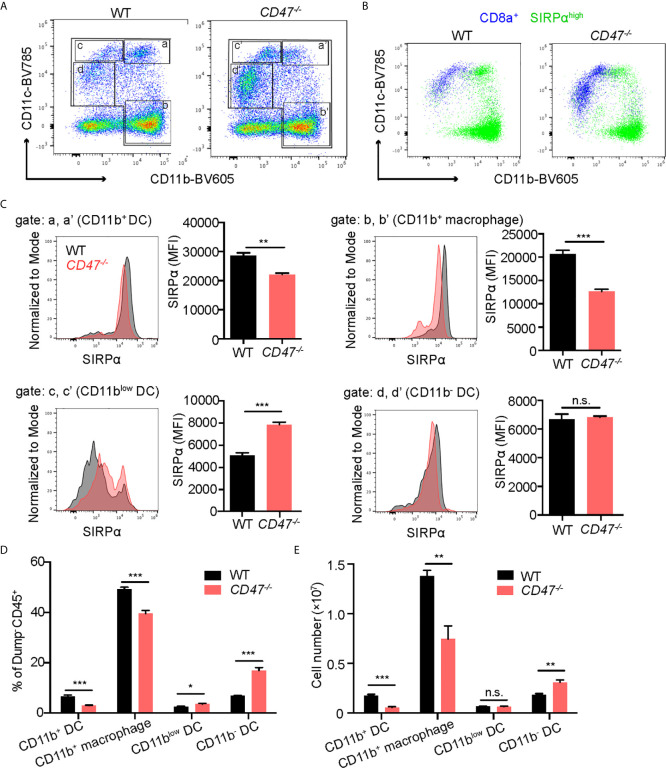
SIRPα expression in CD11b^+^ macrophages/DCs is decreased in CD47 deficient mice with autoimmune uveitis. WT and *CD47^-/-^* mice were immunized and spleen cells were collected 14 days post immunization and examined by flow cytometry. **(A)** Live cells (Zombie UV^-^) excluding a dump gate (T cells; CD3, B cells; CD19, NK cells; NK1.1, and granulocytes; Ly6G) were gated on CD45^+^ cells and further separated by CD11b and CD11c. The gates were defined as follows: Gates a and a’; CD11b^+^CD11c^high^ (CD11b^+^ DCs), gates b and b’; CD11b^+^CD11c^-^ (macrophages), gates c and c’; CD11b^low^CD11c^+^ (CD11b^low^ DCs), and gates d and d’; CD11b^-^CD11c^low^ (CD11b^-^ DCs). **(B)** SIRPα^high^ cells and CD8a^+^ cells in **(A)** are shown in green and blue, respectively. **(C)** SIRPα expression on each macrophage and DC population gated in **(A)** was examined. **(D)** Frequency of each macrophage/DC population in spleens was determined by using frequency of each population in ZombieUV^-^dump^-^CD45^+^ cells. **(E)** Cell number of each macrophage/DC population was obtained by using the number of cells in each SP and the frequency of each population in ZombieUV^-^ cells. Data were analyzed by Student’s *t* test and expressed as mean ± SEM. *P<0.05; **P<0.01; ***P<0.001. n.s., not significant. n=4 mice per group.

CD47 is expressed on homeostatic cells and activates the SIRPα receptor on phagocytes (macrophages and DCs) to suppress engulfment. It has been reported that the lack of CD47 caused diminished expression of its ligand SIRPα and subsequently affects the development of SIRPα^+^ DC cell populations (22, 43). CD11c^high^ DCs are conventional DCs (cDCs), which can be further classified into two subpopulations according to the expression profile of CD8a and SIRPα; CD8a^+^SIRPα^-^ cDCs and CD8a^-^SIRPα^+^ cDCs (44, 45). Consequently, we sought to examine SIRPα expression on phagocytes in our EAU model. Macrophage and DC populations (CD11b^+^ and/or CD11c^+^) in SP cells from 14 days post immunization were analyzed by flow cytometry. We found that the cells with high SIRPα expression were largely found within the CD11b^+^ population regardless of CD11c expression, whereas CD8a^+^ cells were mainly found within CD11c^+^ populations with lower CD11b expression (**Figures 5A, B** **)**. Thus, CD11b^+^ DCs (**Figure 5A**, gate a) mainly consist of CD8a^-^SIRPα^+^ cDCs, while CD11b^low^ DCs (**Figure 5A**, gate c) were mainly CD8a^+^SIRPα^-^ cDCs.

Looking more specifically at SIRPα^+^ populations, SIRPα expression levels in CD11b^+^ cells were decreased in *CD47^-/-^* mice compared to WT mice regardless of CD11c expression: SIRPα expression in CD11b^+^ DCs and CD11b^+^ macrophages (**Figure 5A**, gate b), was lower in *CD47^-/-^* mice (**Figure 5C**). In addition, the cell number and frequency of CD11b^+^ DCs and CD11b^+^ macrophages were decreased in *CD47^-/-^* mice (**Figures 5D, E** **)**. On the other hand, SIRPα expression as well as the frequency and the number of CD11b^low^ DCs (**Figure 5A**, gate c) and CD11b^-^ DCs (**Figure 5A**, gate d), was maintained or rather increased in *CD47^-/-^* mice (**Figures 5C, D** **)**. These results indicate that lack of CD47 leads to diminished SIRPα expression in systemic CD11b^+^ phagocyte populations and consequently leads to impairment in the development of CD11b^+^ DCs and macrophages.

It is known that CD8a^-^SIRPα^+^ cDCs mainly induce CD4^+^ T cell responses, which is an important effector T cell population in autoimmune diseases, whereas CD8a^+^SIRPα^-^ cDCs has greater ability to induce a CD8^+^ T cell response (9, 44, 46). We had identified that CD11b^+^ DCs (corresponding to CD8a^-^SIRPα^+^ cDCs) were decreased in number (**Figure 5E**) and their SIRPα expression profile was also decreased in *CD47^-/-^* mice with EAU (**Figure 5C**). These results indicate that DCs in *CD47^-/-^* mice have decreased ability to induce development of autoreactive CD4^+^ T cell in disease development in response to EAU.

### Macrophage and Dendritic Cell Activation Were Suppressed in CD47 Deficient EAU

**Figure 6:**
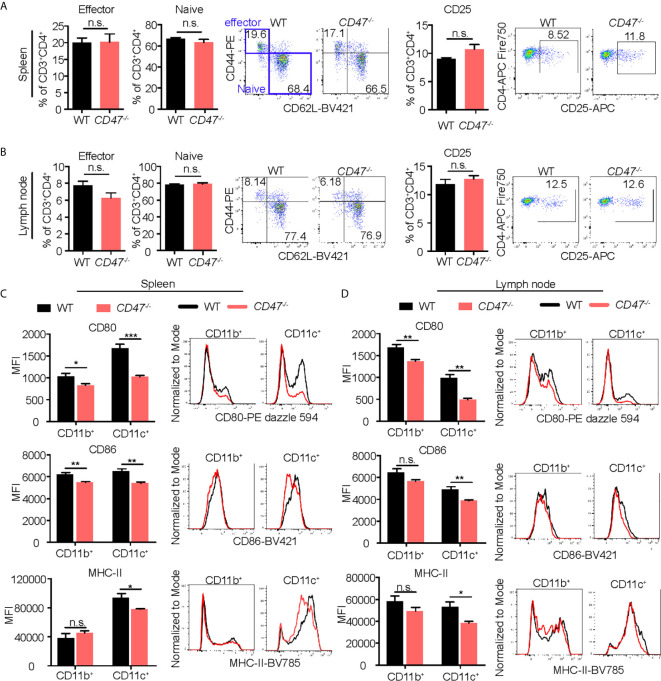
Activation of macrophages and DCs is suppressed in EAU in CD47 deficient mice. WT and *CD47^-/-^* mice were immunized and SP and LN cells were collected 14 days post immunization. Expression of cell activation makers on CD4^+^ T cells, and CD11b^+^ (DCs/macrophages) cells and CD11c^+^ (DCs) cells were evaluated by flow cytometry. **(A, B)** For CD4^+^ T cells, live cells excluding B cell (CD19), NK cells (NK1.1), and granulocytes (Ly6G) were gated on CD45^+^CD3^+^CD4^+^ cells. Frequency of CD62^-^CD44^+^ (effector) cells, CD62^+^CD44^-^ (naïve) cells, and CD25^+^ activated CD4^+^ T cells were examined in spleens **(A)** and lymph nodes **(B)**. **(C, D)** For DCs/macrophages, live cells excluding T cells (CD3), B cells (CD19), NK cells (NK1.1), and granulocytes (Ly6G) were gated on CD45^+^CD11b^+^ and CD45^+^CD11c^+^ cells. Expression of activation markers CD80, CD86, and MHC-II was examined in spleens **(C)** and lymph nodes **(D)**. Data were analyzed by Student’s *t* test and expressed as mean ± SEM. *P<0.05; **P<0.01; ***P<0.001. n.s., not significant. n=4 mice per group.

Given that CD47 has explicit roles in the expression of SIRPα in CD11b^+^ DC/macrophage populations and that the development of CD11b^+^ DC/macrophage populations are impaired in EAU in *CD47^-/-^* mice, we next sought to examine activation markers on systemic immune cells that are crucial in the development of EAU. CD4^+^ T cells in LNs, and DCs (CD11c^+^) and macrophages (CD11b^+^) in SPs were examined for activation markers 14 days after immunization. CD25, CD44 and CD62L were examined in CD4^+^ T cells. CD25 is the interleukin-2 receptor alpha chain present on activated T cells. CD44 is the molecule involved in leukocyte trafficking required for entry into the retina (47). CD62L is a homing receptor expressed on naïve T cells to enter secondary lymphoid tissues and its expression is reduced after activation (48). Effector T cells (CD62L^-^CD44^+^) and naïve T cells (CD62L^+^CD44^-^) along with CD25 collectively represent the activation level of CD4^+^ T cells in EAU (49–52) and various autoimmune diseases (53–55). We found that the frequency between effector and naïve CD4^+^ T cells that were positive for these markers were unchanged in *CD47^-/-^* mice compared to WT mice (**Figures 6A, B** **)**, suggesting that T cell activation does not seem to be significantly affected by loss of CD47.

We next examined macrophage and DC expression of MHC-II, CD80, and CD86; important surface molecules for antigen presentation and co-stimulation required for efficient stimulation of antigen specific CD4^+^ cells. Activated DCs and macrophages increase expression of these molecules and acquire strong T cell priming capabilities (56, 57). Macrophages increase these molecules after activation (58), but to a lesser extent than DCs. We found that all of the macrophage/DC activation markers were decreased in *CD47^-/-^* mice, more strikingly in CD11c^+^ cells (**Figures 6C, D** **)**. These results indicate that DC/macrophage activity related to antigen presentation are impaired in CD47 deficient EAU. Therefore, this functional defect of antigen presentation in *CD47^-/-^* APCs likely caused the suppression of development of CD4^+^ T cells observed in LNs and SPs (**Figure 4B**). However, lack of CD47 didn’t appear to affect the function of CD4^+^ T cells once they were activated given that activation markers in T cells were maintained in CD47*^-/-^* mice (**Figures 6A, B** **)**.

### DC Stimulation Capability of T Cells Was Impaired in CD47 Deficient Mice During EAU; Whereas T Cell Proliferation Was Maintained

Our cell proliferation assay in **Figure 1** demonstrated that the overall immune cell proliferative response against the autoantigen (IRBP) was suppressed in *CD47^-/-^* mice. However, this result represents the proliferative response from total immune cells in the culture, including DCs and macrophages as well as CD4^+^ T cells. As we demonstrated earlier, our flow cytometry results suggested that antigen presenting ability of DCs (CD11c^+^) were significantly affected in *CD47^-/-^* mice, whereas CD4^+^ T cell functions were maintained. Therefore, we next sought to identify which immune cell population, CD4^+^ T cells or DCs, are responsible in suppressing the immune response and subsequent disease severity of EAU in *CD47^-/-^* mice. CD4^+^ T cells in LNs and DCs in SPs isolated by the MACS system were used for mixed lymphocyte proliferation assay. WT and *CD47^-/-^* EAU mice 14 days post immunization was utilized. Isolated CD4^+^ T cells were labeled with CellTrace and cultured with DCs for 72 hours in the presence of IRBP-p. The proliferated CD4^+^ T cell was measured by flowcytometry.

**Figure 7:**
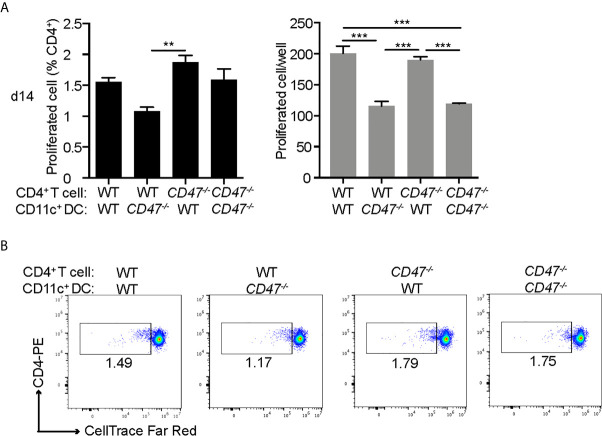
DCs from CD47 deficient mice failed to stimulate T cells in antigen specific response. WT and *CD47^-/-^* mice (n=3-4) were immunized and SP and LN cells were collected 14 days post immunization. CD4^+^ T cells and DCs (CD11c^+^) purified from pooled LN cells and SP cells, respectively, were incubated at a ratio of T cells: DCs =2:1 (2 × 10^5^: 1 × 10^5^/well) in the presence of 10 μg/ml IRBP-p in a 96 well U-bottom plate for 3 days. **(A)** Proliferated cells with reduced expression of CellTrace™ Far Red in live CD3^+^ CD4^+^ cells were analyzed by flowcytometry. **(B)** Representative flow cytometry plots. The samples were run triplicates. Data were analyzed by one-way ANOVA followed by Tukey’s multiple comparison. Data are expressed as mean ± SEM. **P<0.01; ***P<0.001.

The purity of the cells after MACS isolation was more than 94% for CD4^+^ T cells and 99% for DCs (**Supplemental Figure S5**). WT-CD4^+^ T cells and *CD47^-/–^*T cells cultured with WT-DCs (WT-T: WT-DC and *CD47^-/–^*T: WT-DC, respectively) exhibited comparable proliferation, indicating that *CD47^-/^* ^–^CD4^+^ T cells were capable of proliferation with a proper stimulation by DCs. WT-T cells cultured with *CD47^-/–^*DC (WT-T: *CD47^-/–^*DC) had diminished proliferation compared to WT-T: WT-DC, indicating that DCs from *CD47^-/-^* mice were impaired in antigen presentation and T cell stimulation. The proliferation of *CD47^-/–^*T cells cultured with *CD47^-/–^*DC (*CD47^-/–^*T: *CD47^-/–^*DC) was impaired compared to *CD47^-/–^*T: WT-DC but comparable to WT-T: *CD47^-/–^*DC. These results again highlight that antigen presentation and T cell stimulation ability of *CD47^-/–^*DCs are impaired but *CD47^-/–^*T cell proliferative ability is maintained (**Figure 7**).

Together, these results demonstrate that *CD47^-/-^* CD4^+^ T cells maintain their ability to proliferate, which is consistent with our results in **Figure 6**, where we observed that activation markers in *CD47^-/-^* CD4^+^ T were conserved; however, *CD47^-/-^* DCs failed to stimulate both WT and *CD47^-/-^* T cells. This indicates that antigen presenting functions in DCs are impaired in *CD47^-/-^* mice, which is also consistent with our observation in **Figure 6**, in which we demonstrated reduced DC/macrophage activation markers in *CD47^-/-^* mice.

In conclusion, we demonstrated that *CD47^-/-^* mice have reduced ocular inflammation in EAU. It is likely that the lack of CD47 expression in *CD47^-/-^* mice caused reduced expression of its receptor SIRPα specifically on CD11b^+^ APC populations (CD11b^+^ DCs and CD11b^+^ macrophages), resulting in impaired development of these APC populations. In addition, *CD47^-/-^* DC/macrophages express reduced amount of activation markers and *CD47^-/-^* DCs failed to induce CD4^+^ T cell proliferation, resulting in development of reduced number of autoreactive CD4^+^ cell and consequent reduced immune cell infiltration in the retina. Overall our results demonstrated that CD47 plays an important role in development of autoimmune uveitis by promoting SIRPα^+^ DC maturation.

## Discussion

CD47 plays pleiotropic roles depending on the cell type expressing CD47 and its ligands. In this study, we examined the role of CD47 in development of autoimmune uveitis. Our results demonstrated that a systemic loss of CD47 is protective in autoimmune uveitis, whereas local retinal loss of CD47 does not affect disease severity. *CD47^-/-^* CD4^+^ T cells maintained their proliferative ability and activation markers, however, *CD47^-/-^* DCs were hindered in their ability to induce an antigen specific CD4^+^ T cell response. In *CD47^-/-^* mice, DCs and macrophages were decreased in overall numbers as well as key activation markers (CD80, CD86, and MHC-II). The failure in the development of mature CD11b^+^DCs and macrophages, which highly express SIRPα but little CD8α, appears to be a vital mechanism required for EAU induction which is significantly impaired in *CD47^-/-^* mice.

Several studies have demonstrated that the interaction of CD47 with its receptor SIRPα is required for development of SIRPα^+^ DCs (22, 43). It has been reported that SIRPα expression on CD8α^-^ DCs is much greater than that on the corresponding CD8α^+^ DCs and that the number of CD8α^-^ DCs in SIRPα mutant mice was lower than that in wild-type mice (59, 60). Therefore, lack of CD47 can cause insufficient development of CD8α^-^ DCs and decreased expression of SIRPα on these cells, which is likely the reason that *CD47^-/-^* mice had decreased number of CD11b^+^ DCs in EAU.

Although the number of CD4^+^ T cells was decreased in *CD47^-/-^* mice, frequency of CD62^-^CD44^+^ effector T cells and CD25 expression among CD4^+^ T cells were not affected. This implies that CD4^+^ T cell activation by antigen stimulation is maintained once these cells are mature, however, it is likely that the decrease in DC and macrophage number and their insufficient antigen presentation ability resulted in a subsequent decrease in the number of mature T cells. This hypothesis is supported by the result from our mixed lymphocyte proliferation assay in which CD4^+^ T cells from *CD47^-/^* ^-^ mice were able to proliferate when stimulated by DCs from WT mice. These results imply that CD4^+^ T cells from *CD47^-/^* ^-^ are able to proliferate with a proper stimulation. An autoimmune disease model in the brain recently reported the similar findings that *CD47^-/^* ^-^ CD4^+^ T cells were activated and produced even larger amounts of cytokines compared to WT mice (61). Moreover, CD47-SIRPα interaction has bidirectional negative effect on regulation of human T cells and DCs (62) and it has been reported that T cell immune response was enhanced by interruption of CD47 in viral infection (63) and tumor immunity (64). Thus, absence of negative regulation due to lack of CD47 could be the reason that CD4^+^ cells maintain their activation level even with insufficient antigen presenting stimulation by DCs.

In the retina, it has been reported that CD47 is expressed in almost all retinal cells including endothelial cells and a small population of cone and rods photoreceptors also express CD47, however, their expression decreases in EAU (65). Although decreased CD47 expression after EAU induction could be a result of inflammation, endothelial CD47 could play an important role in the mechanism of EAU development. Evidence suggests that CD47-SIRPα interactions also regulate leukocyte adhesion and transmigration to vascular endothelial cells. CD47 expressed on both sides (vascular endothelial cells and leukocytes) could contribute to this mechanism. CD47 on vascular endothelial cells interact with SIRPγ on various leukocytes (66) as well as SIRPα on monocytes (26). However, in our EAU model, when primed WT donor cells were transferred to naïve *CD47^-/-^* recipient mice, which lacks CD47 on retinal vascular endothelial cells, the recipient mice developed comparable inflammation to control WT recipient mice. This implies that local CD47 expression in retinal endothelial cells does not play a major role in transmigration of leukocytes. Indeed, Van et al. demonstrated that CD47 expression is dispensable in the endothelium (67). On the contrary, CD47 on DCs could be a critical factor in controlling migration and efficient initiation of the immune response (12, 59, 67). It has also been reported that CD47 on T cells is an important regulator of LFA-1 and VLA-4 integrins required for their adhesion to endothelial cell ligands, ICAM-1 and VCAM-1 (61). Therefore, it is possible that in addition to the defect in SIRPα^+^ DC and macrophage maturation, defect in adhesion and transmigration of T cells and DCs due to their lack of CD47 might play a role in suppressive mechanism of EAU in *CD47^-/-^* mice.

Complete failure of development of ocular inflammation in an adoptive transfer experiment using *CD47^-/-^* donor cells and WT recipient cells supported our hypothesis that systemic immune response in *CD47^-/-^* EAU is impaired. However, when we interpret the data of adoptive transfer of *CD47^-/-^* mice, we need to be aware that the cells isolated from *CD47^-/-^* mice are at higher risk of being phagocytized. Indeed, it has been reported that LN cells from *CD47^-/-^* mice injected into WT recipient animals are more efficiently phagocytized by macrophages and DCs in recipient mice which express intact SIRPα (68). This could explain why adoptive transfer of *CD47^-/-^* donor cells to WT recipient mice resulted in complete inhibition of inflammation, whereas *CD47^-/-^* mice induced with regular EAU was capable to develop mild inflammation. Similar phenomenon is described in an adoptive transfer model of experimental encephalomyelitis (69).

Microglia are resident immune cells in the retina that can be defined as CD11b^+^ cells with low CD45 expression and share some common function with systemic macrophages and DCs. It is noteworthy that microglia expressed SIRPα at a higher level in *CD47^-/-^* mice compared to WT mice and microglial number were not affected in *CD47^-/-^* mice. In contrast, SIRPα in the systemic CD11b_+_ cells in *CD47^-/-^* mice was decreased and the number of CD11b_+_ cells was also fewer. Although the detailed mechanism needs further investigation, these results imply that retinal microglial SIRPα have a distinct regulation mechanism that does not depend on CD47 expression.

In summary, our data demonstrate that systemic CD47 deficiency is protective for autoimmune uveitis. *CD47^-/-^* mice had reduced inflammation both locally in the eye and systemically. Failure to produce mature CD11b^+^ DCs and macrophages due to their reduced expression of SIRPα appears to be the major mechanism in suppression of EAU in *CD47^-/-^* mice. Since CD47 interacts with multiple receptors and are involved in complex mechanism of immune regulation, various CD47 receptors could be a treatment option for autoimmune uveitis. Of interest, blocking CD47 with CD47-Fc fusion protein has been shown to effectively impair infiltration of Th17 cells into the central nervous system, preventing and resolving EAE (69). Conversely, an additional study that utilized a CD47-Ab failed to suppress the inflammation associated with EAE (70). Given these conflicting studies the route and agent utilized to inhibit the CD47-SIRPa interaction is likely key when considering a therapeutic approach targeting CD47. Thus, further investigation is needed to elucidate the function of CD47 and its receptors and find a possible treatment option in autoimmune uveitis.

## Data Availability Statement

The raw data supporting the conclusions of this article will be made available by the authors, without undue reservation.

## Ethics Statement

The animal study was reviewed and approved by the Animal Care Committee of the Massachusetts Eye and Ear.

## Author Contributions 

KC and YO contributed to the study conception and design. YO, ST, and ML performed EAU and quantification. YO and ST performed flow cytometry. KC oversaw all research related experiments. YO, ST, and KC contributed to the analysis and interpretation of the data. YO, ST, and KC wrote the paper. All authors contributed to the article and approved the submitted version.

## Funding

This study was supported by the National Institute of Health/National Eye Institute: Grants: R01EY031291, R01EY027303 (KC), the Massachusetts Lions Eye Research Fund (KC), and the American Macular Degeneration Foundation Prevention Award (KC).

## Conflict of Interest

The authors declare that the research was conducted in the absence of any commercial or financial relationships that could be construed as a potential conflict of interest.
