# Supplementary figures and images for: CD47 Deficiency Ameliorates Ocular Autoimmune Inflammation
Source: Front Immunol. 2021 May 20;12:680568. doi: 10.3389/fimmu.2021.680568 (PMC8174453; doi:10.3389/fimmu.2021.680568)

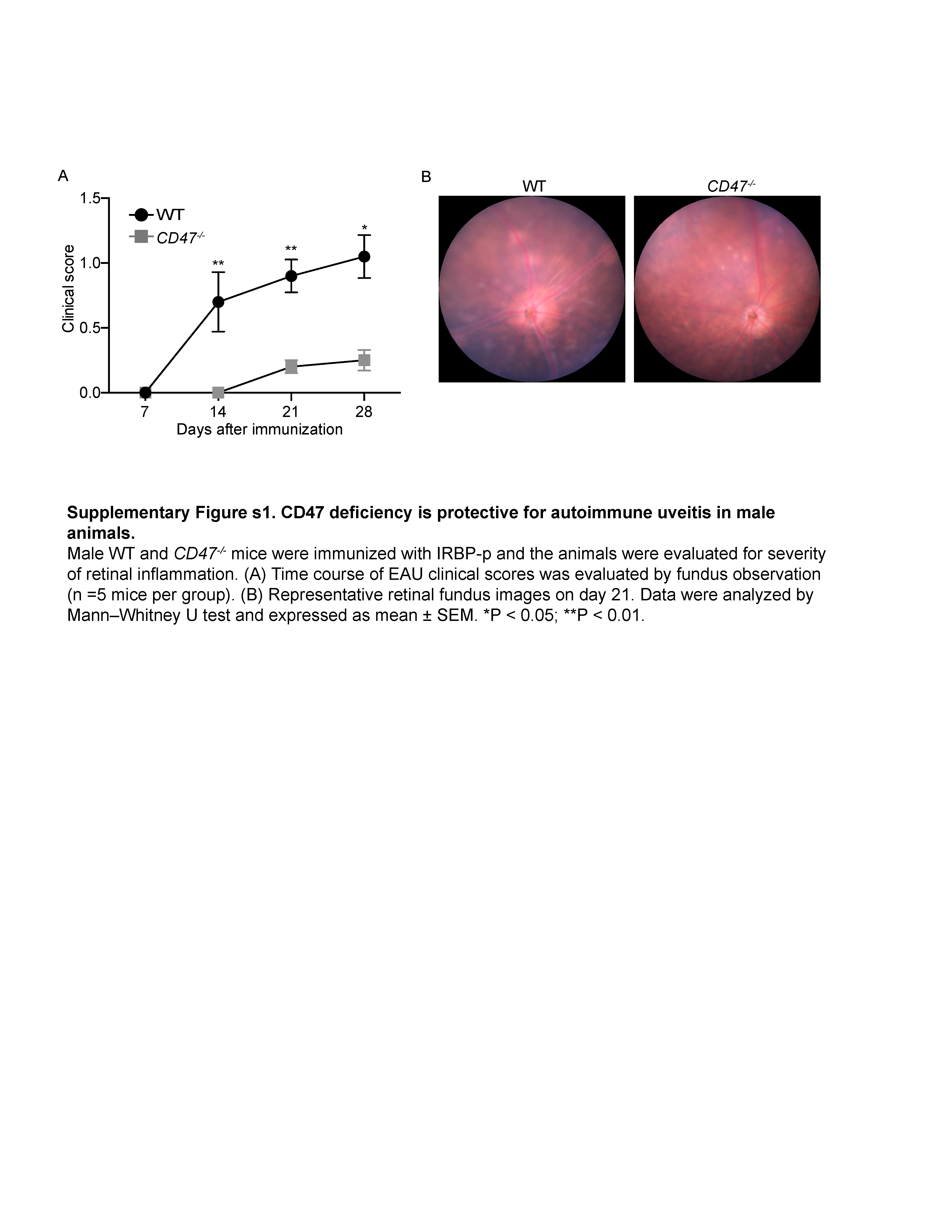

Supplement: Supplementary file 1 [file Image_1.tiff]

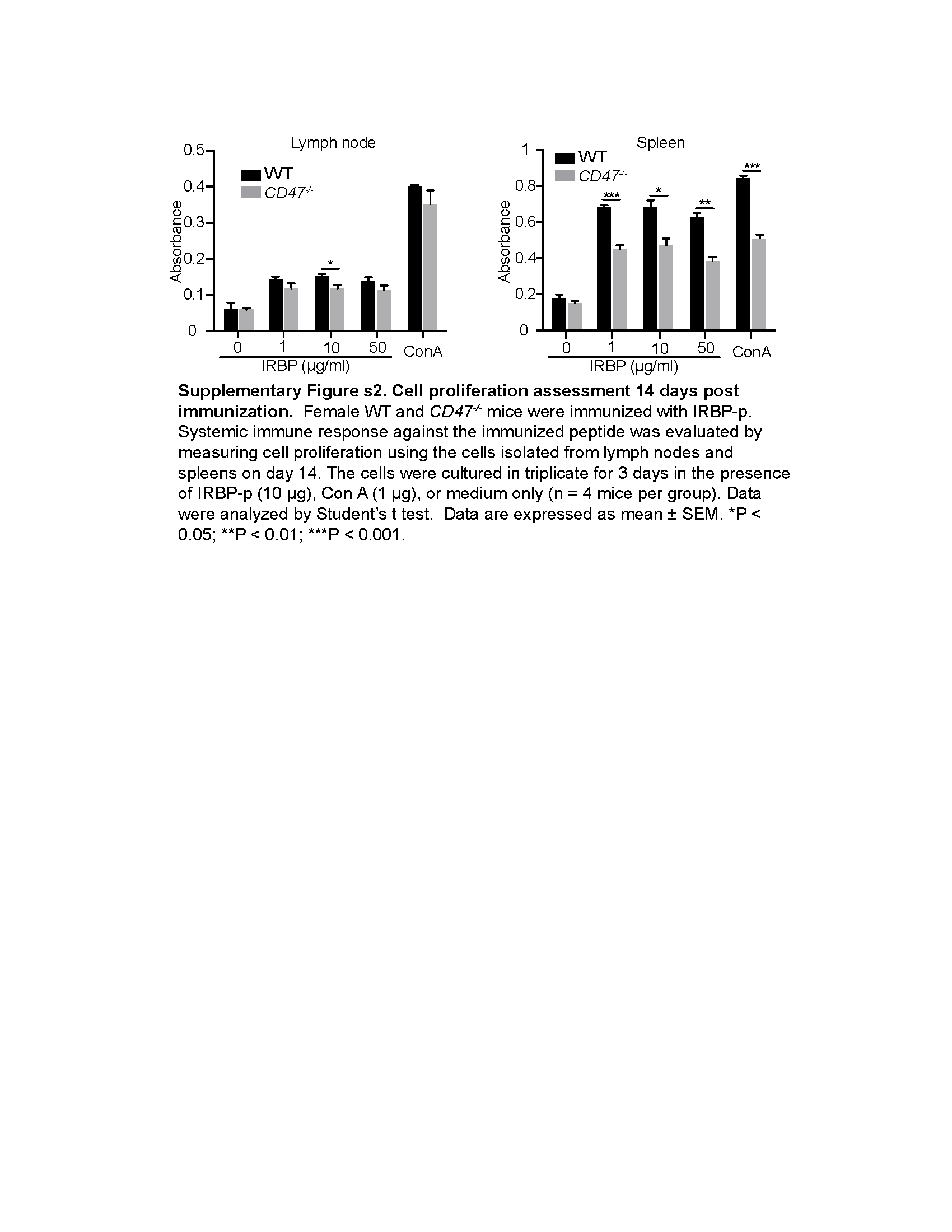

Supplement: Supplementary file 2 [file Image_2.tiff]

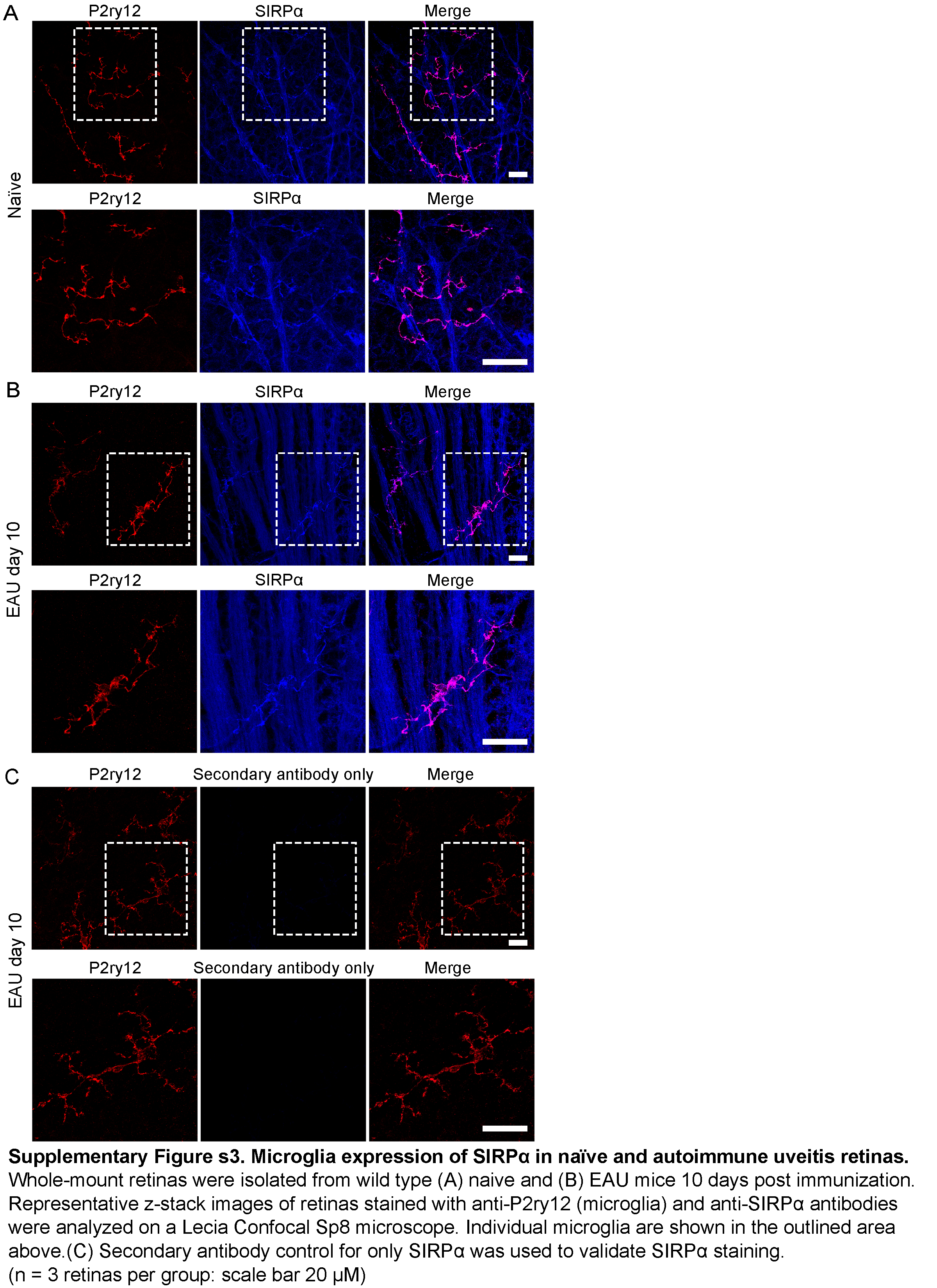

Supplement: Supplementary file 3 [file Image_3.tiff]

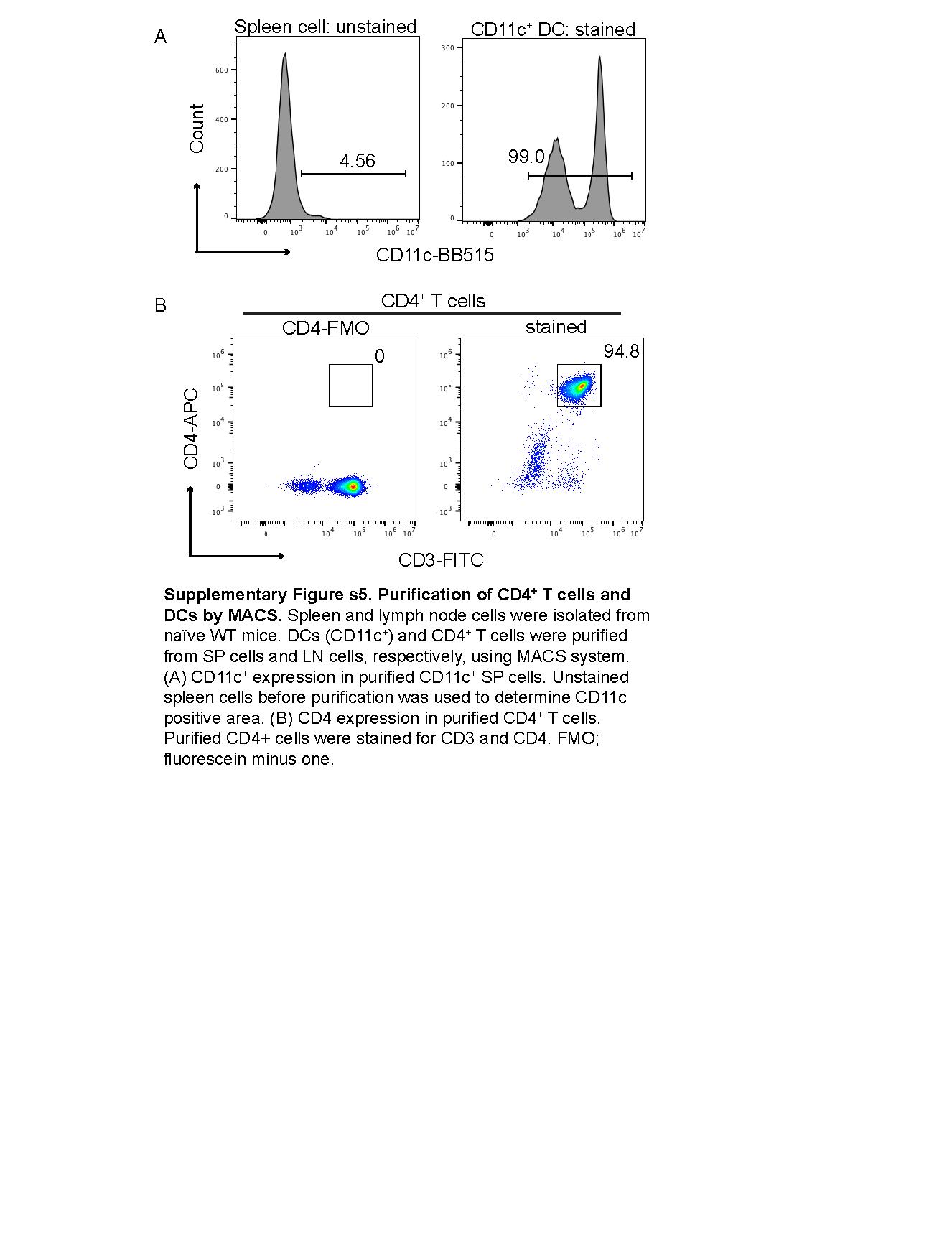

Supplement: Supplementary file 5 [file Image_5.tiff]
